# Polymer Hydrogel Supported Ni/Pd Alloys for Hydrogen Gas Production from Hydrolysis of Dimethylamine Borane with a Long Recyclable Lifetime

**DOI:** 10.3390/polym14214647

**Published:** 2022-11-01

**Authors:** Hao-Kun Cai, Zhong-Yi Jiang, Siyuan Xu, Ying Xu, Ping Lu, Jian Dong

**Affiliations:** 1 College of Chemistry and Chemical Engineering, Shaoxing University, Shaoxing 312000, China; 2 Ningbo Academy of Product and Food Quality Inspection (Ningbo Fiber Inspection Institute), Ningbo 315048, China

**Keywords:** hydrogen production, hydrolysis, dimethylamine borane, polymer hydrogel, Ni/Pd alloy nanoparticles, mechanism

## Abstract

Hydrogen gas production can be produced from dimethylamine borane by the catalytic effect of metal nanoparticles. Past research efforts were heavily focused on dehydrogenation in organic solvents. In this study, hydrolysis of the borane in aqueous solutions was investigated, which bears two significant advantages: that two-thirds of the hydrogen generated originate from water and that the hydrogen storage materials are non-flammable. Polymer hydrogels serve as good carriers for metal particles as catalysts in aqueous solutions. Kinetic analysis of hydrogen production was performed for Ni/Pd bimetallic nanoclusters dispersed in a polymer hydrogel with a 3-D network structure. The reaction catalyzed by the bimetallic nanoclusters has an activation energy of only 34.95 kJ/mol, considerably lower than that by Ni or other metal catalysts reported. A significant synergistic effect was observed in the Ni/Pd bimetallic catalysts (Ni–Pd = 20/1) with a higher activity than Pd or Ni alone. This proves the alloy nature of the nanoparticles in the borane hydrolysis and the activation of water and borane by both metals to break the O–H and B–H bonds. The hydrogel with the Ni/Pd metal can be recycled with a much longer lifetime than all the previously prepared catalysts. The aqueous borane solutions with a polymer hydrogel can become a more sustainable hydrogen supplier for long-term use.

## 1. Introduction

Dimethylamine borane ((CH_3_)_2_NHBH_3_, DMAB) and ammonia borane (AB) can generate hydrogen gas and have become an attractive hydrogen storage material because they serve as liquid hydrogen carriers and release hydrogen on demand [1,2]. DMAB is cheaper and better than NaBH_4_ and AB on the market, with the potential to reduce the cost of hydrogen production. At present, most hydrogen production reactions based on DMAB are carried out in organic solvents such as dioxane, tetrahydrofuran, and toluene [3,4,5,6,7,8,9,10,11,12,13,14,15,16,17,18,19,20,21,22] or via solvent-free dehydrogenation of DMAB [23,24,25]. However, in terms of green energy and safety, it is of critical importance to replace the organic solvents with water. Moreover, 1 mole of DMAB can produce 3 moles of hydrogen by using 2 moles of water (see Equation (1)), compared with the early technology that only 1 mole of hydrogen can be produced in organic solvents from 1 mole of DMAB. Therefore, compared with the organic phase approach, the nonflammable and environmentally friendly aqueous condition can produce more H_2_ from water while consuming the same amount of substrate [26,27,28,29,30,31,32].

(1) $${\left({\mathrm{CH}}_{3}\right)}_{2}{\mathrm{NHBH}}_{3}+2H_{2}O\overset{\mathrm{catalyst}}{\to }\;{\left({\mathrm{CH}}_{3}\right)}_{2}{\mathrm{NH}}_{2}{\mathrm{BO}}_{2}+3H_{2}$$

Recently it has been successfully demonstrated that the above hydrolytic product of BO_2_^−^ metaborate in the aqueous solution can react conveniently with carbon dioxide, resulting in B_4_O_7_^2−^ and CO_3_^2−^ salt, both of which could regenerate borohydride BH_4_^−^ under ambient conditions [33]. This opens a green route for the large-scale application of borane compounds/water as a hydrogen source. This H_2_ production method has great value for portable devices where clean energy is needed from a safe storage material while the catalyst can be reliably reused.

Noble metal catalysts have high catalytic activities for the hydrolysis of borane compounds and the dehydrogenation of such compounds in organic solvents [3,4,5,6,7,8,9,10,11,12,13,14,15,16,17,18,19,26]. However, the scarce reserves and high cost of these precious metal catalysts have limited their applications. In recent years, more attention has been paid to some transition metals, with a high abundance, and their alloys, such as Ni [22,29], Co–Pt nanohybrid [28], Ni/Pd alloy [15], and Ru–Ni nanocomposite [16]. The catalytic activities of single late transition metals are often lower than those of noble metal catalysts and difficult to activate water molecules for splitting the O–H bonds. The combined use of a light transition metal and a noble metal is a proper choice to split water molecules while maintaining the essential activities and driving the cost of hydrogen to a lower level.

To enhance the activities of the metals, the use of catalyst carriers can be a good approach to increase the metal-specific surface area and improve their catalytic stability/activity while maintaining the metals in nanoscale. With the help of the carriers, the metal particles have a lower trend of severe agglomeration during catalysis but retain a high efficiency level. In addition, the separability of the carrier also ensures that the catalyst can be recycled. Therefore, the selection of a proper carrier has a great influence on the catalytic activity and recovery performance.

Polymer hydrogels are ideally suitable for the production of H_2_ gas from the aqueous phase; they can effectively load the metal particles by using their large loading space and functional groups to chelate the metal particles in the gel networks, thus preventing the agglomeration and deactivation of the metals [34]. Hydrogels can be prepared via a hydrophilic polymer material with cross-links, which form networks suitable for both uniform dispersion of the metal ions and chemical reactions in aqueous and non-aqueous solutions.

In this work, Ni/Pd bimetallic nanoparticles in polyacrylamide hydrogel were prepared to catalyze the hydrolysis reaction of the exemplary borane compound, DMAB. The catalytic substrate is extended from the NaBH_4_ in the previous study to dimethylaminoborane (DMAB) in the present study so that the reaction mechanism of hydrogen generation is more comprehensive and the production cost is lower. The synergistic effect and extraordinary recyclability of the catalyst are demonstrated. We also compare kinetics with our previous studies of polymer-supported Ni catalysts. This study represents a step forward for the use of the borane + water solutions as a reliable hydrogen supplier by minimizing the reaction activation energy while dramatically extending the service lifetime of the hydrogel and metals.

## 2. Experimental Section

### 2.1. Materials

Potassium persulfate and hydrochloric acid were purchased from Sinopharm (Shanghai). PdCl_2_, NiSO_4_·7H_2_O, acrylamide, N,N′-methylene bisacrylamide (MBAM), poly(N-vinylpyrrolidone) (PVP, K-30), dimethylamine borane (DMAB) aqueous solution (10.0% wt concentration) and potassium hydroxide were obtained from Aladdin Reagent Company (Shanghai). All reagents of analytical grade were used in the experiments without further purification.

### 2.2. Preparation of Hydrogel-Supported Ni/Pd Nanoclusters

**Scheme 1:**
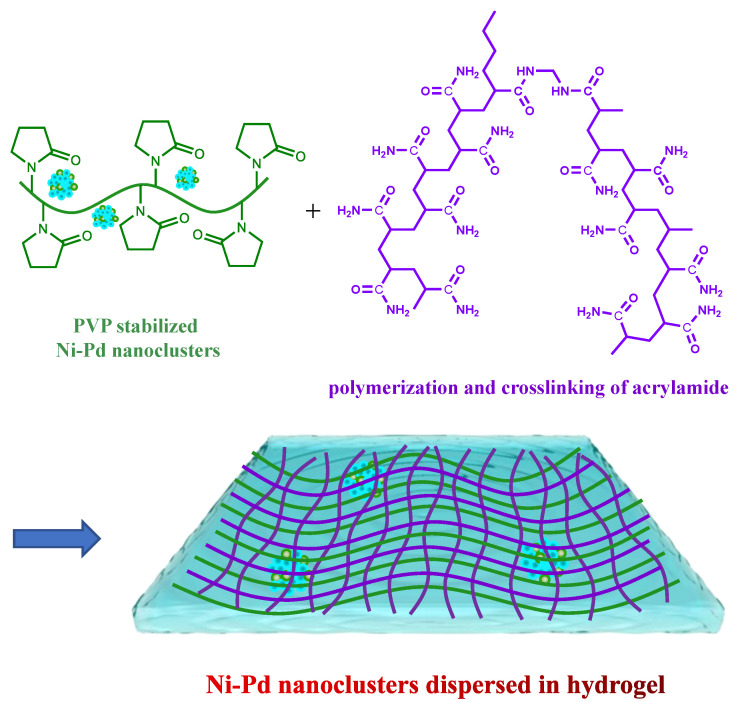
Preparation of Ni/Pd nanoclusters dispersed in the hydrogel consisting of PVP (green lines) and cross-linked poly(acrylamide) (purple lines).

The PVP-protected Ni/Pd colloidal solutions were prepared by adding various amounts of Pd salt into the Ni salt solution and then mixing with acrylamide solution to complete the cross-linking. The preparation method of Ni/Pd nanoclusters in the hydrogels was described in our previous work [34]. Briefly, 2.5 g of PVP-stabilized Ni/Pd colloidal solutions were prepared from the mixtures of varying amounts of PdCl_2_ and NiSO_4_ solutions. NaBH_4_ aqueous solution (0.227 mol/L)was added slowly to the Pd and Ni salt solution while stirring to produce Ni/Pd nanoclusters (Solution A, see Scheme 1). For example, to prepare a Ni/Pd 20/1 solution, 2.5 g of PVP dissolved in 20 mL of water was mixed thoroughly with 20 mL of PdCl_2_–HCl solution (withdrawn from a 200 mL stock solution prepared with 200 mg PdCl_2_ (1.13 mmol) and 0.5 mol L^−1^ HCl solution) and 0.59 g of NiSO_4_·7H_2_O (2.26 mmol) dissolved in 20 mL water. To this Pd/Ni mixture, 0.172 g of NaBH_4_ (4.54 mmol) dissolved in 20 mL of water was added slowly under vigorous stirring until the metals were completely reduced, producing a black Ni/Pd colloidal solution (denoted as Solution A). Subsequently, 7.82 g of monomer acrylamide, 0.93 g of crosslinker MBAM, 0.15 g of initiator potassium persulfate, and 20 mL of water were mixed to prepare Solution B, which was then mixed with Solution A to form a hydrogel via radical polymerization and crosslinking of the acrylamide. The obtained hydrogel was washed in fresh water repeatedly to remove the unreacted compounds and dried in a vacuum oven, giving Ni/Pd nanoclusters dispersed in the hydrogel (Scheme 1) [34]. The mass fraction of the total metal (Ni/Pd = 20/1) in the hydrogel is 0.38%. That of the total metal mass with 1/10 in the hydrogel is 0.27%, with 1/5 is 0.16%, with 1/30 is 0.63%, with Pd is 0.05%, and with Ni is 30%.

### 2.3. Hydrolysis of DMAB Catalyzed by the Hydrogel Supported Ni/Pd Nanoclusters

1.0 g of the hydrogel-supported pure Pd, pure Ni or Ni/Pd nanoclusters with variable Ni/Pd ratios (5/1, 10/1, 20/1, and 30/1) were added to 18 mL of water in a three-neck round bottom flask, which was thermostated in a water bath at 50 °C. Then, 2.0 mL of DMAB aqueous solution (10.0% wt concentration) was added to the flask through a pipette. The volume of H_2_ produced could be recorded by the drainage method with an inverted measuring cylinder. The hydrogen production rate (k) was calculated from the slope of the initial linear part of the curve of the H_2_ volume evolved versus time.

The kinetic activation parameters were determined via the Arrhenius equation and Eyring equation at different temperatures (from 313.15 K to 333.15 K). The experiments were also measured with various amounts of the nanocluster catalyst (0.8, 1.0, and 1.4 g) and different concentrations of DMAB (81, 162, 324, and 486 mM). For the recyclability experiment, the used catalyst was collected by filtration from the aqueous solutions, washed with water three times, and applied to the next round of H_2_ production.

### 2.4. Characterization of Hydrogel-Supported Ni/Pd Nanoclusters

The catalyst was analyzed under a JEM-1230 transmission electron microscope (TEM) at an accelerating voltage of 80 KV using the cryo-ultramicrotomy technique. The nanoparticle sizes were determined by analyzing 50–180 particles from several TEM images and calculating the Feret’s diameters of the nanoclusters using ImageJ software (National Institutes of Health, Bethesda, Md, USA). The standard deviations of the Feret’s diameters were calculated to indicate the particle-size distributions. The concentrations of Ni and Pd in the polymers were analyzed by using an inductively coupled plasma atomic emission spectrometer (ICP-AES) (Teledyne Leeman Labs, Model Prodigy, Mason, OH, USA). A K-ALPHA X-ray photoelectron spectroscopic (XPS) analysis (Thermo Fisher Scientific Company, Waltham, MA, USA) was performed to determine the oxidation state of the Ni/Pd metal. 

## 3. Results and Discussion

### 3.1. Characterization of Ni/Pd Nanoclusters in the Hydrogel

In the preparation of the Ni/Pd nanoparticles, PVP can effectively prevent the aggregation of the metal nanoparticles. In order to reuse the catalyst, polyacrylamide was added into the colloidal solution of the metal nanoparticles, supported by the PVP, to form the crosslinking hydrogels in less than 1 h. The prepared hydrogel containing the cross-linked polyacrylamide and linear PVP prevents the aggregation of the metal nanoparticles.

**Figure 1:**
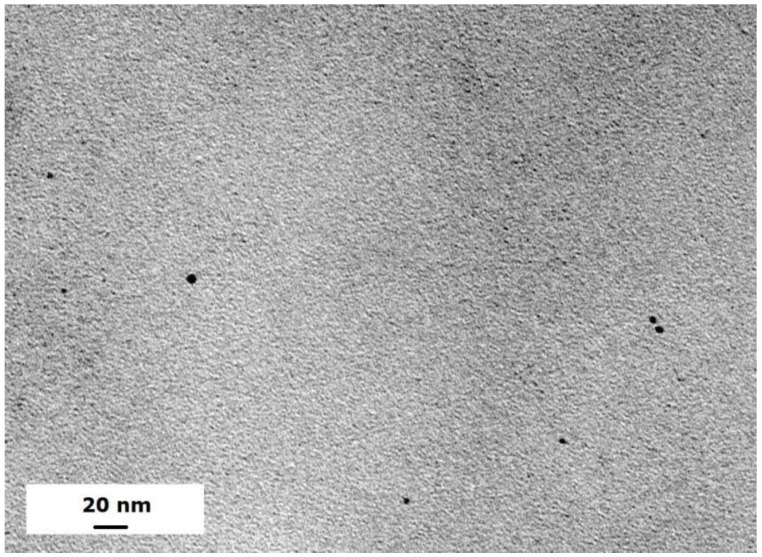
TEM image of metal particles with Ni/Pd with Ni/Pd = 20/1 at a scale bar of 20 nm.

The Ni/Pd metal particles can be uniformly distributed in the hydrogels. Figure 1 shows a typical TEM image of the Ni/Pd particles with Ni/Pd = 20/1 (see black dots in Figure 1). The average sizes of the particles with variable Ni/Pd ratios range from 4.3 to 8.1 nm, as shown in our previous study [34]. The mesh size of hydrogel, measured by the swelling method as described in our previous study [35], is in the range of 2.5–4.3 nm, which is slightly smaller than the size of the metal particles. Thus, the porous hydrogel can firmly fix the metal nanoparticles during reactions.

In order to analyze the content of Pd and Ni in the Ni/Pd alloy supported in the polyacrylamide hydrogel, different radios of Ni/Pd alloys were tested by ICP-AES. As shown in Table 1, when the ratios of Ni to Pd were 5/1 and 10/1, the actual measured value was close to the theoretical value. However, when the ratios of Ni to Pd was 20/1 and 30/1, the nickel content was relatively decreased, which was similar to that of Pd and Co protected by PVP in the literature [36].

The X-ray diffraction curves of the hydrogels with the metals are shown in Figure S1 in Supplementary Materials. The average size of metal crystallites calculated by using the Scherrer formula is 1.06 nm. The average size of the nanoparticles estimated by TEM is from 4.3 to 8.1 nm, exceeding the crystallite size, which is normal due to the fact that the nanoparticles may consist of several crystallites.

**Figure 2:**
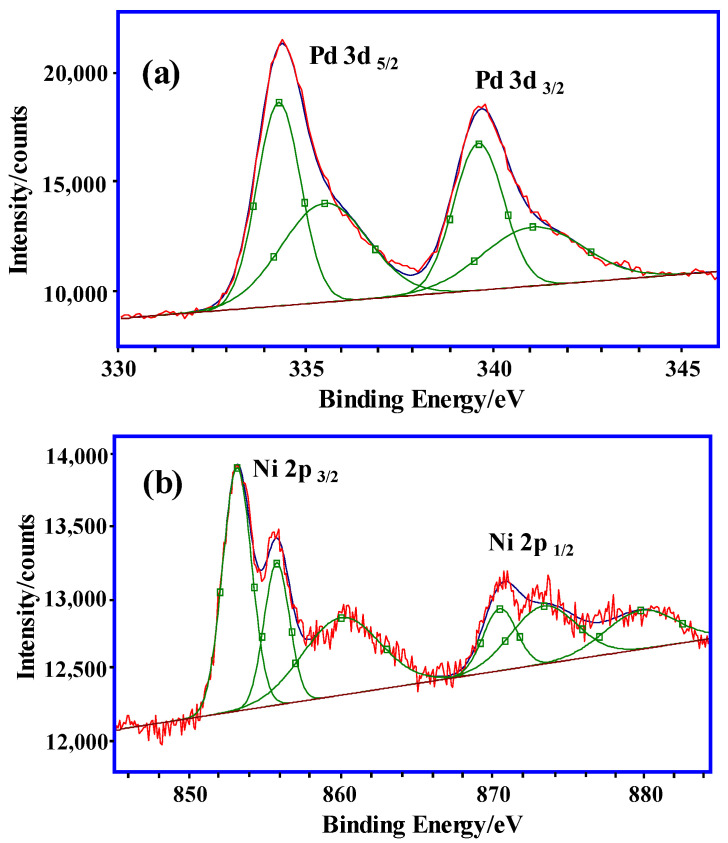
XPS spectrum of (**a**) Pd (3d) region and (**b**) Ni (2p) region in PVP–Ni/Pd (Ni/Pd = 20/1).

For further characterization of the nanoclusters in the hydrogel, the oxidation state of Ni/Pd in the hydrogel was analyzed by XPS. Figure 2a illustrates the XPS spectrum in the Pd (3d) region, which shows that there are two obvious peaks at 334.4 eV and 339.8 eV, which are the d_5/2_ and d_3/2_ electron-binding energies of the Pd at zero oxidation state Pd(0), respectively. This affirms that the Pd is mostly reduced in the prepared catalyst. In Figure 2b, the XPS spectrum in the Ni (2p) region shows that there are two obvious peaks at 853.0 eV and 871.0 eV, which are the 2p_3/2_ and 2p_1/2_ electron-binding energies of the Ni at zero oxidation state Ni(0), respectively. Compared with the standard electron-binding energies of Ni(0) (2p), the Ni 2p_3/2_ peak at 853.0 eV in the prepared catalyst has a binding energy of about 0.4 eV larger, which was due to the decrease in the electron density of the Ni and the increase of the binding energy affected by the Pd. The XPS spectrum indicates that the Ni(0) was difficult to be completely oxidized in the air [37]. Figure 2b shows the broad satellite peak of Ni(2+) 2p_3/2_ at 860.0 eV. There are also obvious peaks at 855.6 eV and 873.1 eV, which are the 2p_3/2_ and 2p_1/2_ electron-binding energies of the NiO, respectively and their peak intensities are weaker than those of the Ni(0). Therefore, the Ni in the hydrogels exists in the form of Ni(0)/NiO, and the content of the Ni(0) is more than that of the NiO. In addition, in the Ni/Pd alloy catalyst, the Ni and Pd atoms could undergo charge transfer and facilitate charge balance while breaking of the O–H bonds and B–H bonds in the adsorbed water and borane molecules to generate hydrogen [31].

### 3.2. Hydrolysis of DMAB Catalyzed by Ni/Pd Nanoclusters in the Hydrogel

**Figure 3:**
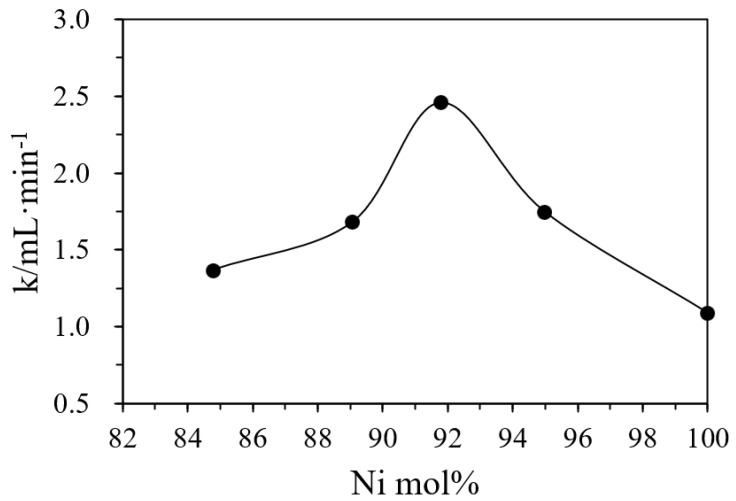
The positive synergy effect in the bimetallic catalysts with varying Ni molar percentages in the total metal amount in the hydrogel determined by ICP-AES on the initial hydrogen production rates. Note that pure Pd nanoparticles in the hydrogel have an initial rate (k) of 1.08 mL/min only.

The monometallic Pd or Ni with activities in catalyzing DMAB [29,38] serve as control samples to examine if they can demonstrate positive synergistic effects (or higher activity) in the Ni/Pd alloys. Ni and Pd alloy metal catalysts are used in this study to catalyze hydrogen production through a combination of different proportions of Ni and Pd. This can greatly reduce the use of the precious Pd metal and further reduce the cost. The activities of the alloy catalysts (Table 2) indicate that the catalysts with different ratios of Ni/Pd indeed had different catalytic activities in the hydrolysis. Both pure Pd and pure Ni showed lower activity and hydrogen production yields. On the contrary, the alloy had higher catalytic activities and H_2_ production rates. The representative curves of hydrogen generation with time are shown in Figure S2 in Supplementary Materials. Table 2 also shows that, with the increase of the nickel content, the value of the rate k also increased and became the highest when the radio of Ni/Pd was 20/1, after which the rate began to decrease with the further increase of the nickel content. In Figure 3, the Ni content of the catalysts with various Ni/Pd ratios, measured by ICP-AES, is plotted against their corresponding rate, which shows that when the nickel content is 91.78%, the rate k reaches the highest value (See Table S1 in Supplementary Materials for confidence intervals of the reaction rates). Thus, the catalyst with Ni/Pd = 20/1 had the best reaction rate.

**Figure 4:**
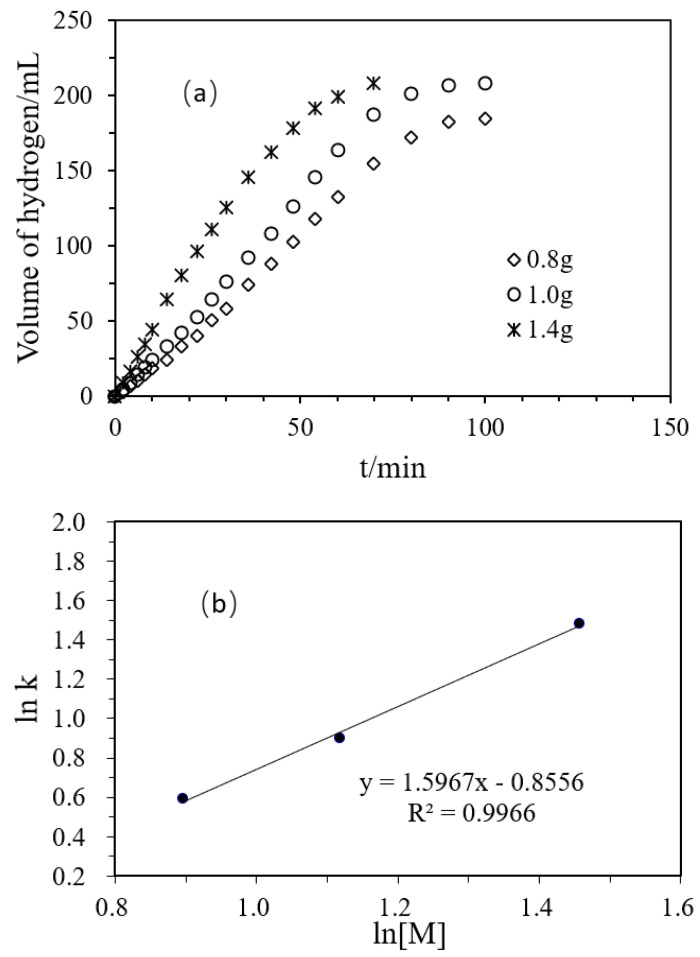
(**a**) Volume of hydrogen versus time plots for the hydrolysis of DMAB catalyzed by Ni/Pd nanoclusters (Ni/Pd = 20/1) with different masses of the hybrid catalyst at 50 °C; (**b**) Plot of the initial hydrogen production rate (k, in mL min^−1^) versus the total concentration of Pd and Ni (in logarithmic scale, y = 1.5967x − 0.8556).

Figure 4a shows changes in the H_2_ production volume with time in the hydrolysis of DMAB, catalyzed by the polyacrylamide hydrogel supported Ni/Pd catalyst (Ni/Pd = 20/1), of different amounts of the catalyst (0.8, 1.0, 1.4 g). In the initial reaction stage, the hydrogen production volume increased with time in a good linear relationship. The figure also shows that 108 mL of hydrogen could be released within 50 min with 0.8 g of the catalyst. When the amount of catalyst was increased to 1.4 g, 183 mL of hydrogen could be released within 50 min. The increase in the catalyst amount could greatly improve the reaction rate under the same conditions. As shown in Table 3, in the absence of the catalyst, the rate of the spontaneous reaction of the DMAB was only 0.21 mL/min and only 1.9% of the yield was obtained after 160 min. On the contrary, hydrogen was rapidly and steadily produced after the addition of the catalyst. So, the catalyst greatly accelerated the reaction. Besides, the initial turnover frequency (TOF) value reached the maximum when the dosage of the catalyst was 1.0 g. In addition, the highest hydrogen production yield of 97.7% was obtained when the catalyst dosage was 1.0 g. Therefore, the following experiments were carried out with the dosage of 1.0 g. Figure 4b shows the logarithmic relationship between the hydrogen production reaction rate and the total concentration of Pd and Ni in the catalyst, and the slope in the illustration was 1.5967.

**Figure 5:**
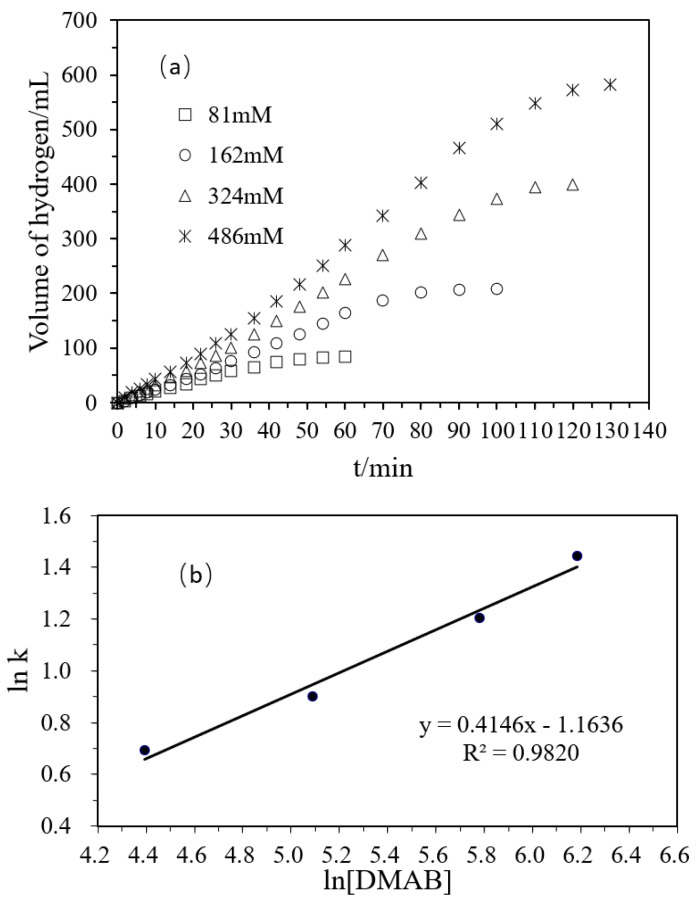
(**a**) Volume of hydrogen versus time plots for the hydrolysis of DMAB catalyzed by Ni/Pd nanoclusters (Ni/Pd = 20/1) (1 g) with different concentrations of DMAB (81, 162, 324, 486 mM) at 50 °C; (**b**) plot of the initial hydrogen production rate (k, in mL min^−1^) versus DMAB concentration (in logarithmic scale, y = 0.4146x − 1.1636).

Figure 5a shows that, with the increase of the concentration of DMAB, the volume of hydrogen generated increased. When the molar concentration ratio of the substrate was 1:2:4:6, the volume ratio of the hydrogen released was 1.00:2.48:4.76:6.93, which was roughly consistent with the initial molar concentration ratio of the substrate. Therefore, with the increase of the substrate concentration, the amount of the hydrogen released increased proportionally. In Figure 5b, the relationship between the hydrogen production rate and the substrate concentration was plotted on a logarithmic scale and the obtained slope was 0.4146. Combined with the above influence of Pd and Ni in the polyacrylamide hydrogel and the concentration of the substrate in the reaction, the rate equation of hydrogen production from DMAB catalyzed by polyacrylamide hydrogel supported Ni/Pd is as follows:

(2) $$\mathrm{Rate}=-\frac{3d[\left({\mathrm{CH}}_{3}{)}_{2}{\mathrm{NHBH}}_{3}\right]}{\mathrm{dt}}=\frac{d\left[H_{2}\right]}{\mathrm{dt}}=k^{\prime }{\left[\mathrm{NiPd}\right]}^{1.5967}{\left[\mathrm{DMAB}\right]}^{0.4146}$$

Previously, we found that, for the hydrolysis of DMAB catalyzed by Ni metal dispersed in the polymer hydrogel, the relationships among the rate, substrate, and metal were as follows [29],

(3) $$\mathrm{Rate}=-\frac{3d[\left({\mathrm{CH}}_{3}{)}_{2}{\mathrm{NHBH}}_{3}\right]}{\mathrm{dt}}=\frac{d\left[H_{2}\right]}{\mathrm{dt}}=k^{\prime }{\left[\mathrm{Ni}\right]}^{1.8064}{\left[\mathrm{DMAB}\right]}^{0.2332}$$

The two rate Equations (2) and (3) show that the hydrolysis reactions with the bimetallic Ni/Pd catalysts are dependent on the DMAB substrate concentrations and the Ni/Pd alloy catalyst in the rate-determining step [32].

A recent study of a Pt/Ni catalyst on carbon showed that the hydrolysis reaction is first-order, with respect to the DMAB and catalyst concentrations [39], as described by Equation (4),

(4) $$\mathrm{Rate}=-\frac{3d[\left({\mathrm{CH}}_{3}{)}_{2}{\mathrm{NHBH}}_{3}\right]}{\mathrm{dt}}=\frac{d\left[H_{2}\right]}{\mathrm{dt}}=k^{\prime }{\left[\mathrm{PtNi}\right]}^{1.3686}{\left[\mathrm{DMAB}\right]}^{0.5196}$$

Our kinetic parameters in Equation (2), very close to those of the Pt/Ni catalyst on carbon in Equation (4), clearly indicate that both Pd and Ni atoms are involved in the activation of the DMAB and water when we compare the above rate Equations (2) and (3).

**Figure 6:**
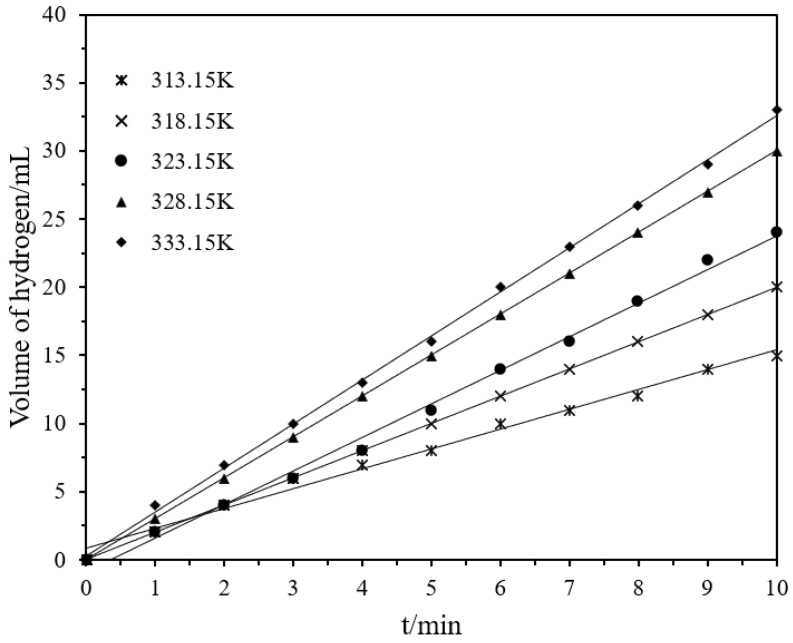
Volume of hydrogen versus time plots for the catalytic hydrolysis of DMAB by Ni/Pd nanoclusters (Ni/Pd = 20/1) (1.0 g) with different temperatures varying from 313.15 to 333.15 K.

In order to determine the influence of temperature on the reaction, the reaction was carried out at different temperatures (313.15~333.15 K) at 162 mM of DMBA concentration. With the increase in temperature, the reaction rate also increased, as shown in Figure 6. The Arrhenius and Eyring equations can be used to obtain the activation energy, enthalpy, and entropy of the hydrogen production by hydrolysis,

(5) $$\ln k=\mathrm{lnA}-\left(\frac{E_{a}}{RT}\right)$$

(6) $$\ln\left(\frac{k}{T}\right)=\ln\left(\frac{k_{B}}{h}\right)+\frac{\Delta S}{R}-\frac{\Delta H}{R}\left(\frac{1}{T}\right)$$

where *T* is the temperature in Kelvin, *R* is the gas constant (8.314 J·mol^−1^k^−1^), E_a_ is the activation energy, *k* is the rate, *h* is the Planck constant (6.626 × 10^−34^ J·s), *k*_*B*_ is the Boltzmann constant (1.381 × 10^−23^ J·k^−1^ ), Δ*S* is the activation entropy, and Δ*H* is the activation enthalpy.

**Figure 7:**
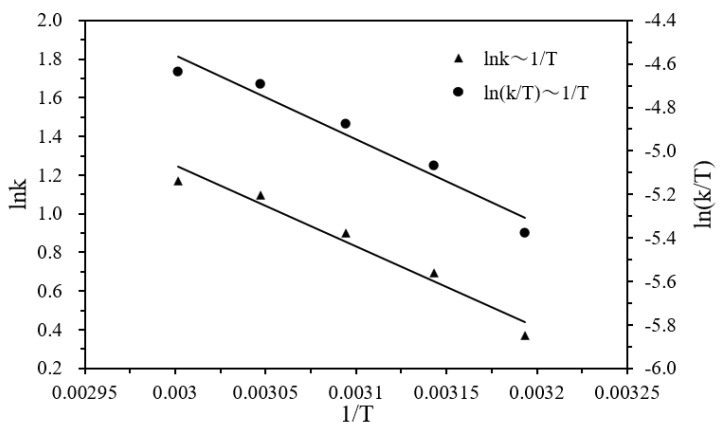
Logarithm of the initial hydrogen generation rate (lnk) versus 1/T plot for the catalytic hydrolysis of DMAB catalyzed by Ni/Pd nanoclusters (Ni/Pd = 20/1) (1.0 g) at various temperatures (solid triangles) and ln(k/T) versus 1/T plot for the catalytic hydrolysis of DMAB (solid circles).

According to Figure 7, the activation energy E_a_, enthalpy ΔH, and entropy ΔS of the hydrogen production were estimated to be 34.95 kJ·mol^−1^, 32.26 kJ·mol^−1^, and −138.64 J·mol^−1^K^−1^, respectively, by using Equations (5) and (6). The negative activation entropy indicates that the total number of molecules was reduced in the rate-determining step of the elementary reaction, and that the hydrolysis reaction proceeded via an associative mechanism in which the participation of the water molecule in the rate-determining step may involve its attack on the DMAB substrate.

Table 4 shows the comparison of activation energy and recyclability (run times) in the hydrolysis of amine borane compounds catalyzed by different catalysts reported previously. The activation energy data of the hydrogel-supported Ni/Pd here is significantly lower than those in the H_2_ production from ammonia borane (AB), ethylenediamine bisborane (EDAB), or dimethylamine borane (DMAB) by other types of metals. The low activation energy (only 34.95 kJ·mol^−1^) as well as enthalpy (32.26 kJ·mol^−1^) in the hydrogel-supported Ni/Pd alloy not only affirms the above-mentioned synergistic effect of the Pd and Ni in the catalyst but also indicates that the energy of the transition state species adsorbed on the alloy metal is lowered significantly in the rate-determining step. This indicates that only a lower barrier energy is required to break H–O bonds in water molecules. The low activation energy is indicative of the hydrogen being produced easily. As far as we know, the activation energy in the present study represents the lowest in a hydrolysis reaction of DMAB so far. Besides, compared with the reactions in the organic phases [3,4,5,6,7,8,9,10,11,12,13,14,15,16,17,18,19,20,21,22] and under solvent-free conditions [23,24,25], the activation energy in the aqueous phase is also lower, indicating that the breaking of B–H bonds occurs smoothly with the attack of water.

We obtained a kinetic Equation (2) of the Ni/Pd catalyst in this study and compared it with that of the Pt/Ni catalyst on carbon in Equation (4) and that of the pure Ni catalyst in Equation (3). They clearly indicate that both Pd and Ni atoms are involved in the activation of the DMAB and water when we compare the rate (Equations (2) and (3)). The two reaction rates (Equations (2) and (3)) show that the hydrolysis reactions with the bimetallic Ni/Pd catalysts are dependent on the DMAB substrate concentrations and the Ni/Pd alloy catalyst concentration in the rate-determining step. If Pd is absent, the rate equation is less dependent on the DMAB concentration when the pure Ni catalyst is used. If Ni/Pd or Pt/Ni is used, the rate equation is more dependent on the DMAB concentration. Thus, more DMAB molecules can be adsorbed on the surface when Pd atoms are present. The reaction involves the activation of the substrate by the metals acting in synergy, due to an even lower activation energy in the bimetallic catalyst than that in the Ni metal alone or the Pd metal alone (see entries 10, 15, and 16 in Table 4).

**Scheme 2:**
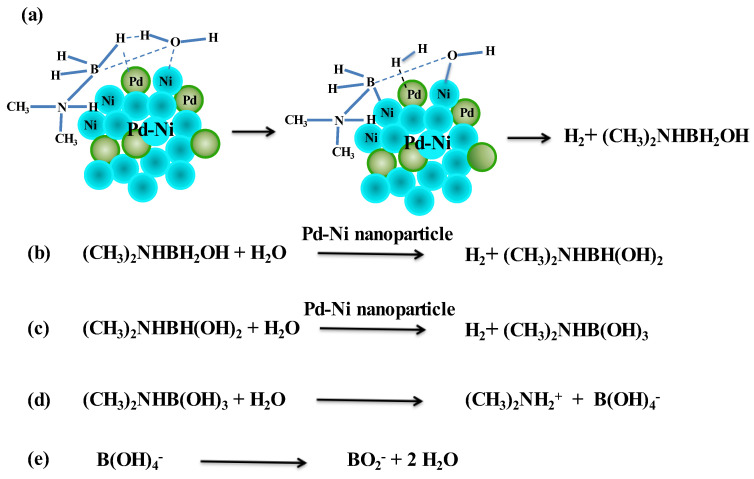
Proposed mechanism for hydrolysis of DMAB catalyzed by bimetallic nanoparticles.

A bimetallic reaction mechanism based on our experiments is postulated as follows. The adsorption of DMAB and water on the Ni/Pd nanoparticles involves the elimination of H_2_, forming the hydroxylated intermediate, (CH_3_)_2_NHBH_2_^+^OH^-^ (see Step (a) in Scheme 2). The breaking of H–O and B–H bonds may occur simultaneously with the formation of H_2_ on the metals. This step may arise from the activation of water on the metal surface and the substitution for a hydrogen atom in the borane moiety via the attack on the DMAB substrate by the OH group of the water [51]. This is supported by the above measurement result of the negative activation entropy of the reaction. In Step (b) of Scheme 2, the water and (CH_3_)_2_NHBH_2_OH adsorbed on the Ni/Pd surface are activated again, resulting in the breaking of the O–H and the second B–H bond and formation of the second H_2_ molecule and (CH_3_)_2_NHBH(OH)_2_. In Step (c) of Scheme 2, the hydrolysis of (CH_3_)_2_NHBH(OH)_2_ gives rise to the third H_2_ molecule and (CH_3_)_2_NHB(OH)_3_, which can be hydrolyzed to (CH_3_)_2_NH_2_^+^ and B(OH)_4_^−^ (Step d). The latter can dehydrate to produce the metaborate BO_2_^−^. The total reaction corresponds to that in Equation (1) (see above). 

The recent studies of bimetallic catalysts for ammonia borane (AB) indicated that a low activation energy 36.9 kJ·mol^−1^ was obtained in a PdCo alloy, supported on ZIF-derived N-doped carbons [52], and that of 34.2 kJ mol^−1^ in NiCu bimetallic nanoparticles [53]. These findings are consistent with our results that the activation of the key intermediates to split water and borane molecules efficiently occurs with bimetallic catalysts. The use of monometallic Ni or Co to activate water is not efficient enough. The two methyl groups in DMAB increase the electron density on the N atom and may help to stabilize the (CH_3_)_2_NHBH_2_^+^ intermediate in step (a) in Scheme 2. 

### 3.3. Reusability of the Hydrogel Supported Ni/Pd Nanoclusters in the Hydrogen Production from DMAB

**Figure 8:**
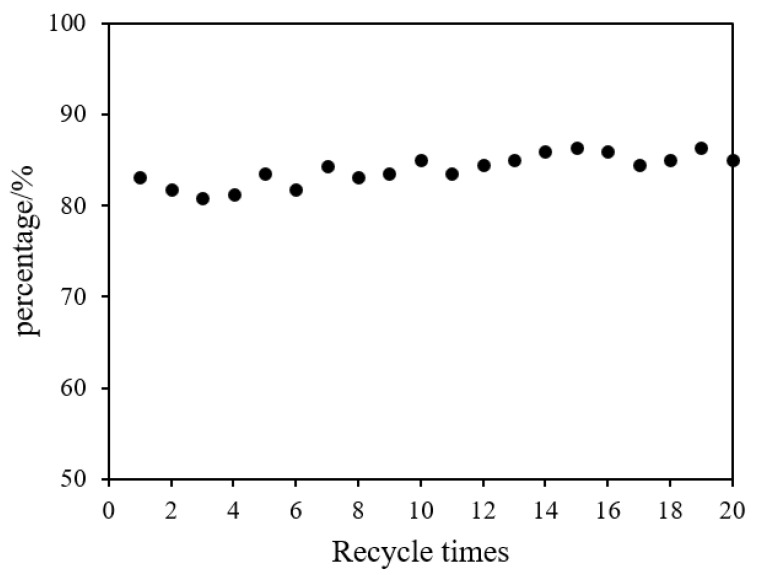
The percentage of catalytic activity versus recycle times for the hydrolysis of DMAB by Ni/PdNi–Pd nanoclusters (Ni/Pd = 20/1) (1.00 g) at 50 °C.

Figure 8 shows the recycling performance of polyacrylamide hydrogel supported Ni/Pd metals in the catalytic hydrolysis of DMAB. Hydrogen (208 mL) could be obtained within 95 min when the catalyst was used for the first time. In its second service, the used water-DMAB was replaced by a fresh water solution of DMAB, and the hydrogel-metal was reused; the system could produce 177 mL hydrogen in the same time, which was 85% of the initial activity. In Figure 8, from the second re-run and afterwards, the volume of hydrogen released within 95 min was stable at 178.5 ± 6.5 mL, which was about 85% of the initial activity. Remarkably, after 20 times of the recycling test, 181 mL of hydrogen could still be released within 95 min, which is 87% of the initial activity. Therefore, the polyacrylamide hydrogel-supported Ni/Pd catalyst can maintain an extremely long usable life, for at least 20 times, compared with the other catalysts shown in Table 4. The cost of hydrogen generation can be further lowered significantly by extending the lifetime of the metal catalysts.

The polyacrylamide hydrogel-supported Ni/Pd catalyst has a good recovery performance mainly because of the three-dimensional, cross-linked hydrogel in which the metal particles are firmly bound in the hydrogel. However, metal leaching is possible in the catalytic reaction. In order to calculate the leaching amount of metal in the catalyst after the first catalytic reaction, 1 mL was removed from the reacted solution and the volume was fixed to 10 mL for ICP-AES detection. The result shows that, after the first catalytic reaction, the leaching amount of metals in the catalyst was 0.13% of the total metal content in the catalyst, which was quite low. The leaching of metal may lead to the slow decline of the catalyst activity.

## 4. Conclusions

The present work provides a sustainable energy technology for hydrogen production with an aqueous solution of DMAB catalyzed by a hydrogel-supported Ni/Pd catalyst. We show here that the production is improved by taking full advantage of water activation and the cost can be lowered by using water as the H_2_ source, a catalyst with an enhanced lifetime. DMAB has a lower price than AB. The bimetallic particles dispersed in the hydrogel exhibit good activity. The activation energy is only 34.95 kJ·mol^−1^, which is the lowest in the hydrolysis reaction of DMAB, as far as we know. A reaction mechanism is revealed to account for the hydrolysis reaction, including the negative entropy, kinetic equations, and synergistic effect of both the metals. Due to the effective protection by the three-dimensional hydrogel network, the metal particles only experience a negligible loss during the recycling and can be conveniently recycled for at least 20 times. The vast majority of the metal catalysts used here is the low-cost Ni, with a content of 92% in the bimetallic catalysts, and which is expected to boost the hydrogen economy. This efficient, low-cost, and reusable catalytic method can be applied to the preparation of other kinds of bimetallic nanoparticles embedded in hydrogel for the reaction.

## Figures and Tables

**Table 1:** Actual metal contents in the hydrogel and particle average diameters.

	Pd	Ni/Pd = 5/1	Ni/Pd = 10/1	Ni/Pd = 20/1	Ni/Pd = 30/1	Ni
actual molar ratio	-	5.57/1	8.14/1	11.17/1	18.92/1	-
Ni mol%	0	84.78	89.06	91.78	94.98	100

**Table 2:** Hydrogen production yields and rates (k) by hydrogel catalysts with various ratios of Ni/Pd at 50 °C.

	Pd	Ni/Pd = 5/1	Ni/Pd = 10/1	Ni/Pd = 20/1	Ni/Pd = 30/1	Ni
V (mL)	109	209	210	208	180	106
Yield (%)	51.2	98.1	98.6	97.7	84.5	49.8
k (mL/min)	1.08	1.37	1.68	2.46	1.75	1.09

**Table 3:** Effect of catalysts (Ni/Pd = 20/1) amount for the hydrolysis of DMAB.

m (g)	0	0.8	1.0	1.4
k (mL/min)	0.21	1.81	2.46	4.40
Initial TOF (h^−1^)	-	90	96	94
V (mL)	4	184	208	208
Yield (%)	1.9	86.4	97.7	97.7
Reaction time (min)	160	100	95	70

**Table 4:** Comparison of the E_a_ and recyclability for the hydrolysis of amine borane compounds ^a^.

Entry	Catalyst	Amine Borane Compound	E_a_ (kJ/mol)	Recyclability Runs	Ref.
1	CoRh@PVP nanoclusters	AB	42.7	5	[40]
2	NiCo alloy on N-doped carbon	AB	43.6	6	[41]
3	Co-Mo-B on Ni foam	AB	43.6	-	[1]
4	Pd0/CeO_2_	AB	68.00	5	[42]
5	Ru(0) nanoparticles	AB	69.00	5	[43]
6	Ru/C	AB	76.00	-	[44]
7	Ni in p(AAc-co-VI)	EDAB	47.48	-	[45]
8	Pd@p(SPA)	EDAB	67.79	10	[46]
9	Pd/C	DMAB	-	-	[47]
10	Ni-hydrogel	DMAB	50.96	8	[29]
11	Pt-Ni on carbon	DMAB	39.79	5	[39]
12	Co/MoS_2_	DMAB	58.31	5	[48]
13	RhCo nanoclusters	DMAB	50.3	5	[49]
14	Ru(0) nanoparticles	DMAB	92.4	-	[50]
15	Pd/porous carbon	DMAB	60.4	5	[38]
16	Ni/Pd in hydrogel	DMAB	34.95	20	This work

^a^ Note that data about dehydrogenation in organic solvents are not listed here. AB: ammonia borane; EDAB: ethylenediamine bisborane; DMAB: dimethylamine borane.

## Data Availability

Not applicable.

## Supplementary Materials

The following supporting information can be downloaded at: https://www.mdpi.com/article/10.3390/polym14214647/s1, Table S1. Confidence Intervals of Reaction Rates, Figure S1. X-ray diffraction results of the hydrogels with various metal nanoparticles: and Figure S2. Representative volumes of hydrogen gas produced from Ni/Pd 30/1 with time over 160 min and the initial hydrogen volumes in the first 10 min.
