# Supplementary material for: Polymer Hydrogel Supported Ni/Pd Alloys for Hydrogen Gas Production from Hydrolysis of Dimethylamine Borane with a Long Recyclable Lifetime
Source: Polymers (Basel). 2022 Nov 1;14(21):4647. doi: 10.3390/polym14214647 (PMC9656576; doi:10.3390/polym14214647)
Supplement: Supplementary file 1 [file polymers-14-04647-s001.zip › polymers-1937934-supplementary.pdf]

Article

Polymer Hydrogel Supported Ni/Pd Alloys for Hydrogen Gas Production from Hydrolysis of Dimethylamine Borane with a Long Recyclable Lifetime

Hao-Kun Cai [1,2], Zhong-Yi Jiang [2], Siyuan Xu [1], Ying Xu [1], Ping Lu [1] and Jian Dong [1,*]

[1] College of Chemistry and Chemical Engineering, Shaoxing University, Shaoxing 312000, China

[2] Ningbo Academy of Product and Food Quality Inspection (Ningbo Fiber Inspection Institute), Ningbo 315048, China

* Correspondence: jiandong@usx.edu.cn; Tel.: +86-575-88342511

Table S1. Confidence Intervals of Reaction Rates

Different samples in Figure 3 have different confidence intervals.

Sample	pure Pd	Ni/Pd 5:1	Ni/Pd 10:1	Ni/Pd 20:1	Ni/Pd 30:1	pure Ni
reaction rate (ml/min)	1.08	1.37	1.68	2.46	1.75	1.09
standard deviation (ml/min)	0.03	0.04	0.04	0.06	0.03	0.03
95% confidence interval (ml/min)	1.01-1.15	1.29-1.46	1.59-1.78	2.34-2.59	1.68-1.83	1.02-1.17

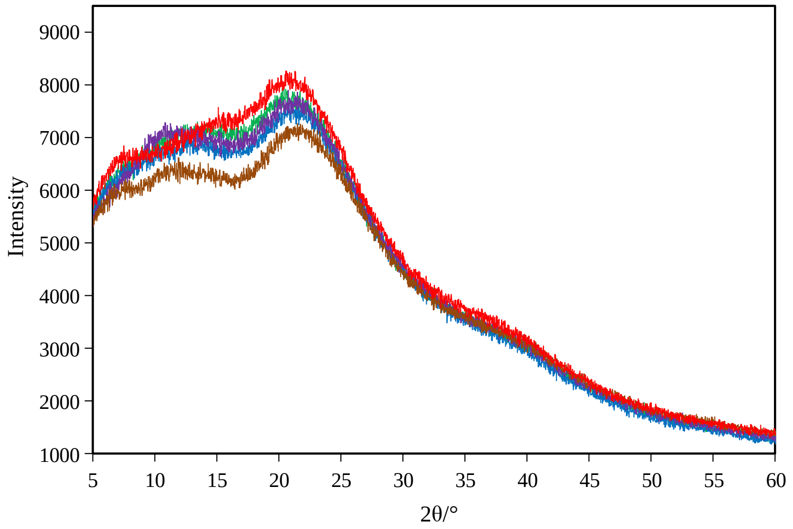

Figure S1. X-ray diffraction results of the hydrogels with various metal nanoparticles: (brown) pure Pd; (blue) PdNi with Pd/Ni=1/5; (purple) Pd/Ni =1/10; (green) Pd/Ni =1/20; (red) Pd/Ni =1/30.

The average size of metal crystallites are calculated by using the Scherrer formula,

$$D=K\lambda/(\beta \cos \theta),$$

where D is the crystallite grain size of the particle (nm), K =0.94.

The wavelength of X-ray λ=1.5418 A. $\Theta = 2\theta/2$.

β = the full width at half maximum intensity of the peak (in rad).

The samples all show broad diffraction peaks with 2θ=22.65°, β=8°=0.1397. Thus, the average size of metal crystallites D=1.06 nm.

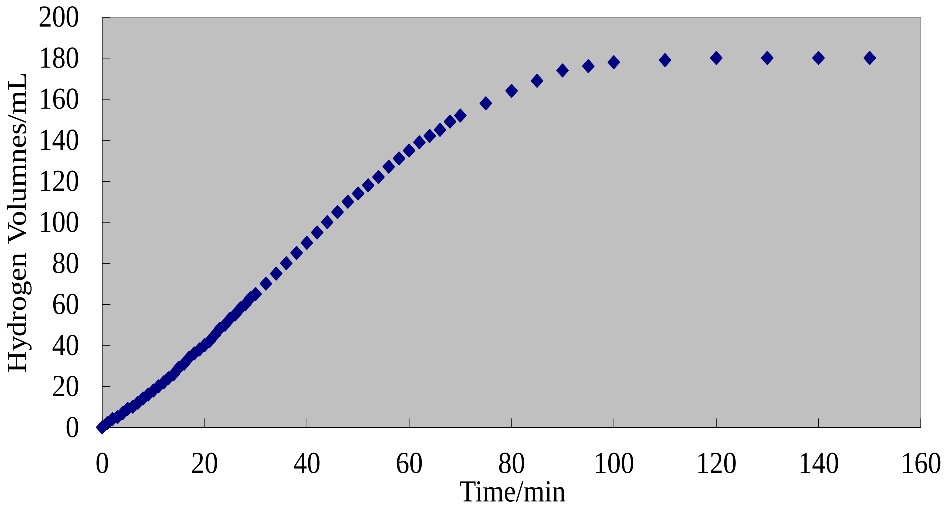

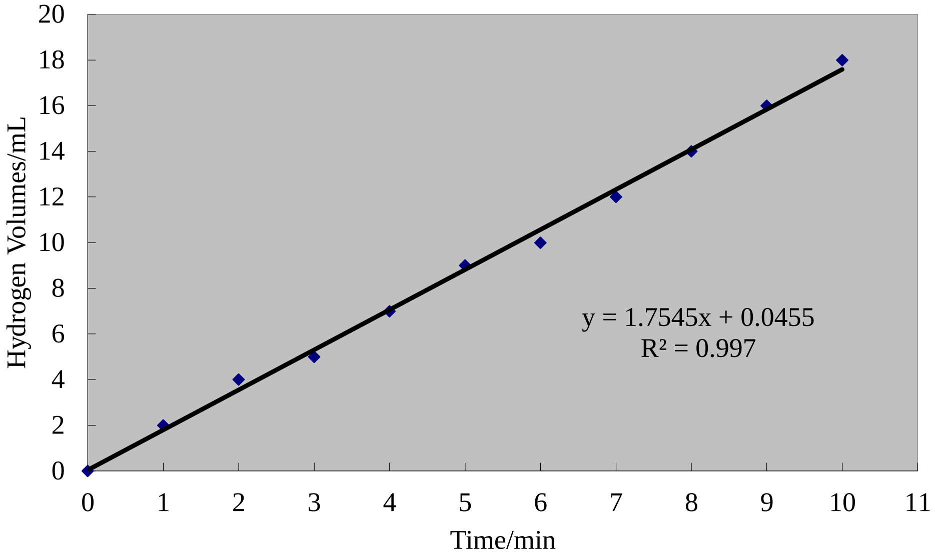

Figure S2. Representative volumes of hydrogen gas produced from Ni/Pd 30/1 with time over 160 min and the initial hydrogen volumes in the first 10 min.
